# Comparative Finite Element Analysis of Stress Concentration in Class V Lower Premolar Cavities Differently Restored by Adhesive Dental Materials

**DOI:** 10.1055/s-0045-1814776

**Published:** 2026-01-20

**Authors:** Shakhawan Kadir Kadir

**Affiliations:** 1 College of Dentistry, Hawler Medical University, Kurdistan Region, Iraq

**Keywords:** Activa BioActive-Restorative, Cention40, finite element analysis, glass ionomer cement, strain, stress distribution

## Abstract

**Objective:**

This study aimed to evaluate the stress distribution of different restorative materials in Class V restorations under varying occlusal loads using finite element analysis.

**Materials and Methods:**

A three-dimensional model of the mandibular first premolar was prepared using computer-aided design (version 4.0 SR8, United States), MIMICS, and 3-MATIC software (3-Matic Medical 13.0, Materialise NV, Belgium). The ANSYS 16.0 (2020) program was used to calculate and evaluate the displacement and stress distribution under four different applied forces at the buccal cusp tip (0.4 mm, right angles), ranging from 100 to 250 N in a restored Class V cavity using (glass ionomer cement [GIC], Activa BioActive-Restorative, and Cention40).

**Results:**

The stress values in the unrestored tooth increased progressively with the applied load, ranging from 49.23 MPa at 100 N to 123.15 MPa at 250 N. Stress concentrations in the GIC restoration were lower compared with the unrestored tooth at all load levels, with values starting at 35.00 MPa at 100 N and rising to 87.51 MPa at 250 N. Among the tested materials, Cention40 composite exhibited the lowest stress concentrations especially at 100 N (28.02 MPa), suggesting better performance under high-load conditions. Activa BioActive-Restorative showed favorable stress distribution but had slightly higher stress values compared with the other materials.

**Statistical Analysis:**

Statistical analysis showed no significant differences in stress distribution among the materials ( *p* = 0.202). There were statistically significant differences between loads in terms of stress ( *p* = 0.004). Stress values increased significantly with higher occlusal loads for all groups ( *p* < 0.05).

**Discussion:**

All the restorative materials exhibited comparable stress distribution patterns. Load intensity is the dominant factor influencing stress distribution in Class V restorations.

**Conclusion:**

Load intensity was the dominant factor influencing stress distribution in Class V restorations, while the type of restorative material played a secondary role.

## Introduction

Dental caries is a prevalent and preventable condition that, in untreated cases, can lead to toothache, tooth loss, and gradual damage to tooth tissue. 1 Consequently, choosing appropriate restorative materials is crucial, as it aids in reducing biofilm, caries formation, periodontal disease risk, and stress on dental tissues. 1 2 Improper stress distribution and biofilm accumulation can lead to restoration detachment and leakage. 3 4 Polymerization shrinkage stress plays a significant role in the long-term success of adhesive restorations, influencing marginal integrity and overall restoration durability. 5

Teeth and supporting structures are exposed to chewing and biting forces continuously, as a result this creates stresses within the tooth. 6 The long-term performance of restorative materials is critically influenced by their mechanical compatibility with the tooth structure. 7 Consequently, numerous studies have aimed to evaluate mechanical behavior, polymerization shrinkage, and stress distribution in restored teeth, which play significant roles in their functional durability. 8 9 10 11

The quality and performance of dental restorations are shaped by the physical and chemical properties of the used material, as these impact the pattern of stress distribution and reduce the stress concentration. 12 13 Restoration durability and the strength of surrounding tooth tissue are significantly related to the elastic modulus of the material; a similar elasticity to dental tissue ensures perfect stress distribution, but achieving this is challenging, as enamel and dentin have different elastic properties. 14 15 Numerical-based methods such as finite element analysis (FEA) provide applicable computerized methods and software to understand, calculate, predict, and evaluate strain, stress distribution, and deformations in target restorative materials. 6 16 17 18

Dentists have used many restorative materials over time including amalgam, gold, ceramics, composite resins, and resin-modified glass ionomer cements (RMGICs), each of which has its advantages and disadvantages. 19 20 21 Amalgam and gold are biocompatible but rarely used for cosmetic reasons; ceramics may trap plaque at restoration margins. 22 23 Resin-based dental composites are polymeric materials and remain the preferred choice for direct restoration of decayed teeth. 24 Alternative restorative options include glass ionomer cements (GICs), RMGICs, and compomers. 25 GICs remain central in pediatric dentistry due to their biocompatibility and fluoride-releasing, remineralizing properties. 26 Newer bulk-fill materials and alternatives such as Cention N aim to reduce shrinkage stress and improve performance in deep posterior cavities through improved monomers and ion-releasing bioactive fillers. 27

An acid–base dental material, such as GIC, is created through the reaction between weak polymeric acids and aluminofluorosilicate glass. 28 GIC is widely used in procedures such as restorations, luting, cavity lining, and root caries treatment as a result of its capacity to bond to teeth, match color, and release fluoride. 28 29 Recently, Activa BioActive-Restorative (Pulpdent, United States) was launched as a bioactive material that unites the strength and esthetics of composites with the benefits of glass ionomers, serving as a hybrid of RMGIC and resin composite. 30 31 It is a novel bioactive dental material that mimics the physical and chemical characteristics of natural teeth by combining bioactive fillers, an ionic resin matrix, and a shock-absorbing resin component. 31 The alkaline restorative material Cention40 is based on urethane dimethacrylate and contains glass fillers that release hydroxide, calcium, and fluoride ions, giving full-volume restorations a high density of polymer networks. 32 33

The analysis was performed to evaluate von Mises stress and analyze stress distribution patterns in Class V dental restorations from three distinct restorative materials under occlusal forces ranging from 100 to 250 N. Using FEA, the research investigated the link between observed stress behaviors and the properties of the tested materials.

*Null hypothesis* : There is no difference in stress distribution among the restorative materials (GIC, Activa BioActive-Restorative, and Cention40) used in Class V restorations, and variations in occlusal load have no significant effect on the stress patterns.

## Materials and Methods

### Study Design and Setting

The research was conducted in Erbil, situated in the Kurdistan Region of Iraq, from December 2024 to March 2025. Ethical approval for the experimental protocol was granted by the ethics committee at College of Dentistry, Hawler Medical University, ensuring alignment with the ethical standards established in the Declaration of Helsinki. A three-dimensional (3D) finite element model of the mandibular first premolar was developed and analyzed to simulate biomechanical responses.

### 3D Model Construction and Cavity Preparation

The 3D model of the mandibular first premolar was obtained from the Sketchfab database, originally created by the School of Dentistry, University of Dundee. The model was first processed using computer-aided design (CAD) software (Rhinoceros 4.0 SR8, McNeel North America, Seattle, Washington, United States).

The CAD model was imported into MIMICS (Materialise NV, Belgium) for preprocessing. Floating pixels were corrected, and a smoothing filter was applied to improve geometric accuracy. The tooth model was then segmented and prepared for meshing.

A standardized Class V cavity was digitally created on the buccal surface with the following dimensions:

- Mesiodistal width: 3 mm
- Occlusogingival height: 2 mm
- Depth: 1.5 mm

The occlusal margin was placed in enamel, and the gingival margin extended into dentin. All internal line angles were rounded to reduce stress concentration.

*Adhesive layer simulation* : A uniform adhesive layer of 20 µm was assumed along all cavity walls to represent clinical bonding conditions, perfectly bonded to both the tooth and restorative material.

### Meshing and Finite Element Model Preparation

The processed 3D model was imported into 3-MATIC (3-Matic Medical 13.0, Materialise NV) for mesh refinement. The working area was clipped, smoothed, and the mesh topology was corrected using the Fix Wizard tool.

A tetrahedral 4-node element mesh was created, with refinement at the cavity and restorative interfaces to ensure accurate stress calculation. The final mesh included ∼220,000 elements and 310,000 nodes. Figs. 1 and 2 illustrate the model and mesh structure.

**Fig. 1:**
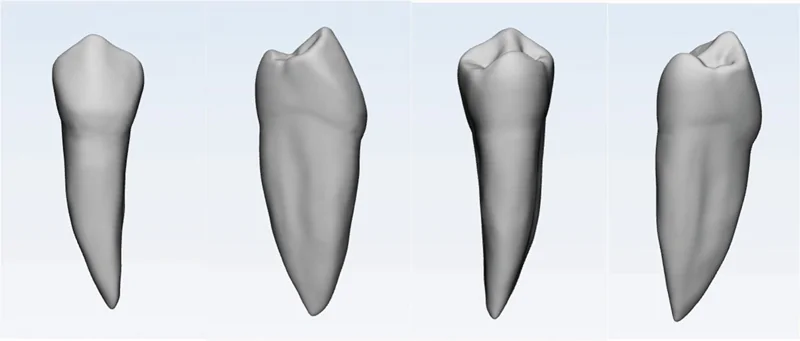
Model of the mandibular premolar from different perspectives.

**Fig. 2:**
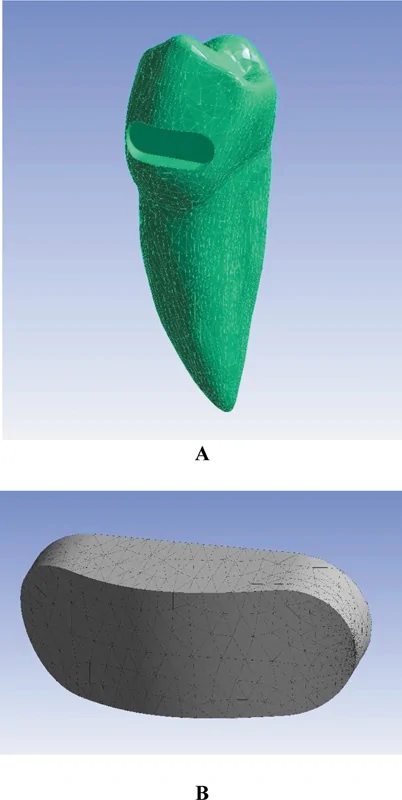
Virtual model after meshing. (A) Mandibular premolar with prepared cavity, (B) restored material, and (C) bone block.

### Material Properties

Materials simulated included enamel, dentin, bone, and three restorative materials: GIC, Activa BioActive-Restorative, and Cention40. All materials were modeled as linearly elastic, homogeneous, and isotropic. The elastic modulus and Poisson's ratio for each material are listed in Table 1 .

**Table 1:** Biomechanical parameters of dental tissues and anatomical regions incorporated in the finite element analysis

Materials	Modulus of elasticity (MPa)	Poisson's ratio (μ)	Reference
Enamel	84,100	0.33	29
Dentin	13,700	0.31	29
Glass ionomer cement	10,800		29
Activa BioActive-Restorative	2,350	0.25	30
Cention40	13,000	0.3	29

### Loading and Boundary Conditions

Occlusal loads of 100, 150, 200, and 250 N were applied perpendicularly 0.4 mm from the buccal cusp tip to simulate lateral excursive forces. The base of the bone block was fully constrained to prevent displacement. The occlusal boundary was positioned within the enamel layer, while the gingival margin extended into the dentin. To avoid stress concentration, the cavity internal line angles were designed to be rounded. 34

### Stress Assessment

The von Mises stress criterion was used because it allows comparison of different materials under complex loading and provides a single equivalent stress value. It is particularly appropriate for ductile materials such as composites and GICs. Stress distribution, total deformation, and concentration zones were calculated using ANSYS APDL 16.0 (ANSYS Inc., Pennsylvania, United States), focusing on cavity walls, restoration margins, and adjacent tooth structures.

## Results

### Stress Distribution in Mandibular Premolars

The stress distribution in mandibular premolars with a Class V cavity, both without restoration and with different restorative materials, was evaluated under varying occlusal loads applied at the buccal cusp ( Figs. 3 4 5 6 ). The von Mises stress values in megapascals (MPa) for each condition are presented in Table 2 . The stress values in the unrestored tooth increased progressively with the applied load, ranging from 49.23 MPa at 100 N to 123.15 MPa at 250 N. Stress concentrations in the GIC restoration (A2) were lower compared with the unrestored tooth (A1) at all load levels, with values starting at 35.00 MPa at 100 N and rising to 87.51 MPa at 250 N. The Activa BioActive-Restorative (A3) showed improved stress distribution, and its stress concentrations remained higher than those of the other restorative materials. Among the restorative materials tested, Cention40 dental composite (A4) exhibited the lowest stress concentrations, especially at 100 N (28.02 MPa).

**Table 2:** von Mises stress in mandibular premolars under varying loads

Applied load (N)	A1 (MPa)	A2 (MPa)	A3 (MPa)	A4 (MPa)
100	49.23	35.00	47.10	28.02
150	73.88	52.50	70.66	42.03
200	98.51	70.01	94.21	56.04
250	123.15	87.51	117.77	70.06

Note: A1, without restoration; A2, glass ionomer cement; A3, Activa BioActive-Restorative; A4, Cention40.

**Fig. 3:**
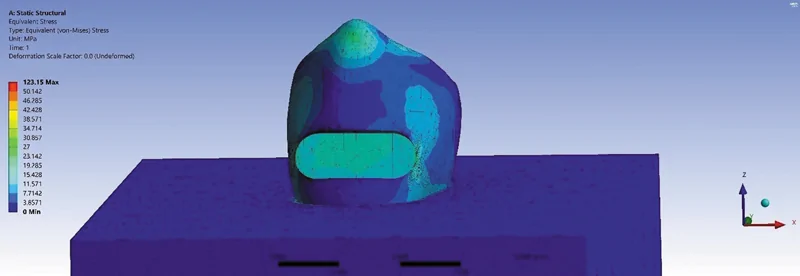
Stress distribution in the tooth without restoration, 250 N.

**Fig. 4:**
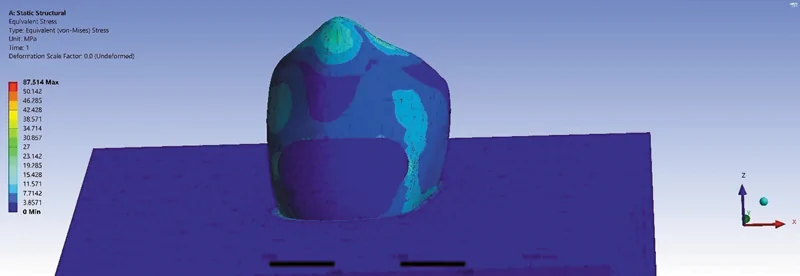
Stress distribution in the GIC-restored tooth, 250 N.

**Fig. 5:**
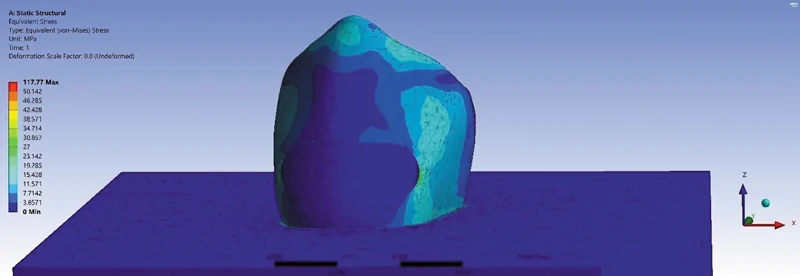
Stress distribution in the bioactive-restored tooth, 250 N.

**Fig. 6:**
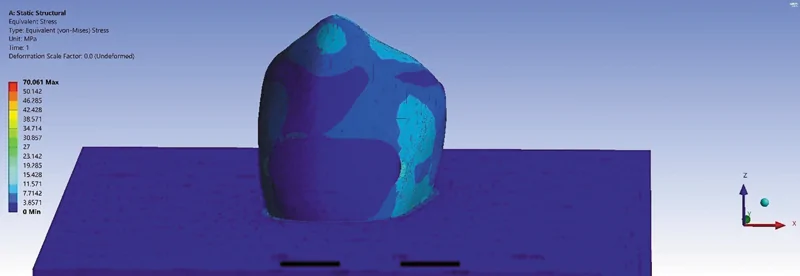
Stress distribution in the Cention40-restored tooth, 250 N.

### Stress Distribution by Restoration Type and Load Levels

Statistical analysis (analysis of variance [ANOVA]: *F* = 1.792, *p* = 0.202) revealed no statistically significant variation in stress levels among the three restorative material groups. This suggests that all materials performed similarly in terms of stress distribution.

On the other hand, the ANOVA test revealed a significant difference in stress values between the different load levels (100, 150, 200, and 250 N) ( *F* -statistic: 7.72, *p* -value: 0.004), with higher loads resulting in greater stress concentration.

### Compare Stress Values at Different Load Levels (Paired *t* -Tests)

Statistical analysis was performed to further explore the differences in stress values at various load levels, as shown in Table 3 . According to the tests, a significant difference in stress was observed at various load levels ( *p* < 0.05) for all materials.

**Table 3:** Comparison of stress values at different loads

Pair	Mean difference	*t* -Value	Difference	*p* -Value
100 vs. 150 N	24.94	5.42	3	0.012
100 vs. 200 N	3.76	5.38	3	0.013
100 vs. 250 N	37.16	5.42	3	0.012
150 vs. 200 N	−21.18	−5.42	3	0.012
150 vs. 250 N	12.22	5.42	3	0.012
200 vs. 250 N	33.40	5.42	3	0.012

## Discussion

Cervical lesions, particularly Class V lesions, are common and challenging to restore due to their location and the complex biomechanical environment. 35 Premolars are especially prone to these lesions because occlusal forces are concentrated at the cervical region, potentially leading to enamel and dentin disruption. 34 36 37 38 FEA allows detailed assessment of internal and marginal stress distributions in such restorations, providing insights not easily obtainable through *in vitro* or clinical studies alone. 39

Additionally, restorations in the mandibular arch have a higher failure rate than those in the maxillary arch due to the lingual orientation of the mandibular teeth, which concentrates tensile stresses at the cervical area, especially in the premolars, leading to failure under stress. 40 41

In this study, FEA using ANSYS 16.0 (2020) was performed to assess von Mises stress and distribution in Class V restorations (mandibular first premolar) for three materials under occlusal loads of 100 to 250 N. Forces were applied perpendicularly 0.4 mm from the buccal cusp tip, aligning with standard biomechanical protocols. 42 43 44

In this study, separate layers for enamel and dentin were used. This unique feature of FEA was usually neglected in previous studies. Additionally, investigation was performed to determine the distribution of stress in various parts of the tooth based on the biomechanical properties of used materials such as the modulus of elasticity and the Poisson's ratio, which help ensure more predictable clinical outcomes, as outlined by Rajagopal and Sharma. 45 Moreover, numerous studies have highlighted that the characteristics of dental materials significantly influence stress patterns in teeth restored under various occlusal forces. 34 35 39 41 46 47 Thus, this study evaluated the von Mises stress in Class V restorations of three different restorative materials, GIC, Activa BioActive-Restorative (Pulpdent), and Cention40.

The previous study used a human mandibular first molar with a Class II cavity restored with EverX Posterior (elastic modulus: 11.4 GPa) and Activa Bioactive (elastic modulus: 2.35 GPa) materials, applying a 600-N static occlusal load at a 60-degree angle. FEA results showed that Activa Bioactive, with its lower elastic modulus, absorbed more stress within the material itself, leading to higher deformation and stress concentration compared with EverX Posterior, which had a higher elastic modulus. 47 In comparison, the present study found that while Activa Bioactive, with its lower elastic modulus (2.35 GPa), showed improved stress distribution, its stress concentrations were still higher than those of other restorative materials, such as Cention40 dental composite, which exhibited the lowest stress concentrations at 100 N. A study by N et al 34 evaluated stress distribution in Class V restorations of mandibular premolars restored with microfilled composite, flowable composite, GIC, and RMGIC. They applied occlusal loads of 100, 150, 200, and 250 N to the restored cavities using finite element modeling. The study found that GIC exhibited von Mises stress values ranging from 36.0 MPa at 100 N to 90.1 MPa at 250 N. In comparison, the present analysis also showed GIC's stress concentrations, with values starting at 35.00 MPa at 100 N and rising to 87.51 MPa at 250 N. While both studies observed similar trends in stress concentration, with GIC showing moderate stress levels, the results of this investigation highlight that Cention40 exhibited the lowest stress concentrations across all applied loads compared with GIC. Another study by Pai et al 41 evaluated stress distribution in Class V cervical lesions of mandibular premolars restored with Cention N, GIC, and dental amalgam, which differed in elastic modulus, with Cention N having the lowest, followed by GIC and amalgam having the highest (35,000 MPa). In the study, occlusal pressure loads of 100, 150, 200, and 250 N were applied to the restored cavities, and it was found that GIC had the lowest von Mises stress values. Cention N showed stress concentrations similar to GIC, while amalgam had the highest stress values. However, these results differ from ours, where Cention40 showed the lowest stress concentrations across all applied loads, outperforming GIC. The difference in stress patterns is mainly due to variations in FEA modeling, including the use of separate enamel and dentin layers, the inclusion of an adhesive interface, and the unique properties of Cention40's Isofiller, which reduce polymerization shrinkage and better redistribute occlusal forces compared with previous studies.

The ANOVA test revealed no statistically significant difference in stress values among the three restoration groups ( *F* -statistic: 1.792, *p* -value: 0.202), indicating that the materials performed similarly in terms of stress distribution. However, significant differences were found between load levels ( *F* -statistic: 7.72, *p* -value: 0.004), with stress increasing as the load increased. Additionally, the paired *t* -tests showed that changes in applied load significantly affect von Mises stress values. It is important to note that most FEA studies do not use statistical analysis to assess these differences.

*Null hypothesis evaluation* : The null hypothesis, which stated that the type of restorative material would not affect stress distribution, is partially rejected. Although statistical differences were not significant between materials, the observed variations in stress concentrations and deformation patterns indicate that material properties, particularly elastic modulus, influence stress behavior in Class V restorations under occlusal loading.

## Limitations

The use of only three restorative materials and a single mandibular first premolar model might not accurately represent the variety of clinical settings. Additionally, von Mises stress was the only focus of the analysis, and other factors such as wear, material fatigue, and long-term performance in clinical settings were not taken into account.

## Conclusion

- Load intensity is the primary factor affecting stress distribution in Class V mandibular premolar restorations, while material type plays a secondary but relevant role.
- Cention40 demonstrated the lowest stress concentrations, suggesting superior mechanical compatibility with tooth structures compared with GIC and Activa BioActive-Restorative.
- FEA effectively allowed assessment of internal and marginal stress distribution, highlighting regions prone to mechanical disruption, which cannot be easily observed in experimental studies.
- The null hypothesis was partially rejected: Material properties influence stress patterns, with higher or compatible elastic modulus materials better distributing occlusal forces in Class V cavities.
- The study provides novel insights by quantitatively linking restorative material properties, cavity geometry, and occlusal load to stress distribution in cervical restorations, supporting informed clinical decision-making.
